# Supplementary material for: Association between oral microbiome and five types of respiratory infections: a two-sample Mendelian randomization study in east Asian population
Source: Front Microbiol. 2024 Apr 10;15:1392473. doi: 10.3389/fmicb.2024.1392473 (PMC11039966; doi:10.3389/fmicb.2024.1392473)
Supplement: Supplementary file 1 [file Data_Sheet_1.zip › Supplementary Materials/MR plots of saliva/Pneumonia/g__unclassified_mgs_2172/leave_one_out.pdf]

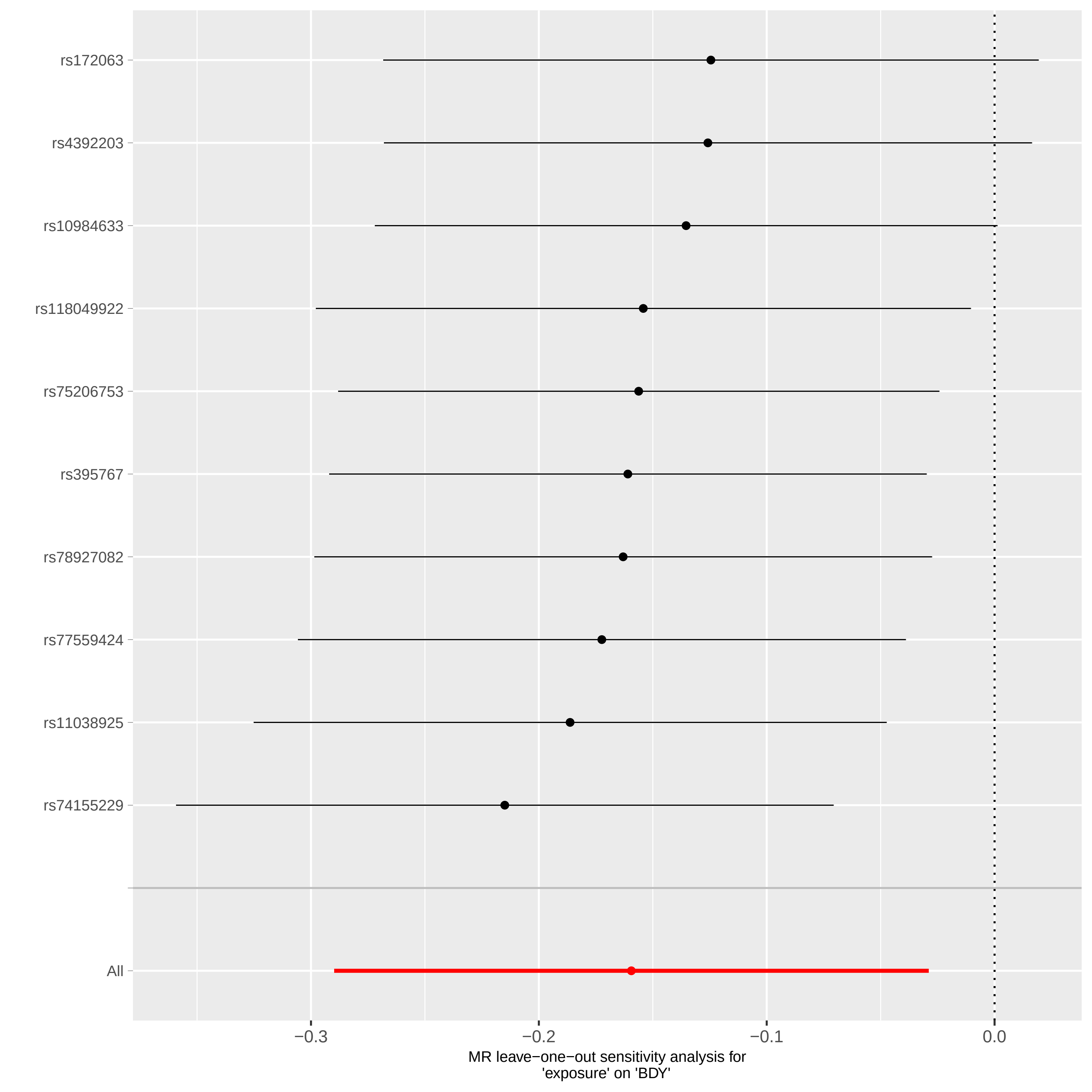

rs172063
rs4392203
rs10984633
rs118049922
rs75206753
rs395767
rs78927082
rs77559424
rs11038925
rs74155229
All
−0.3
−0.2
−0.1
0.0
MR leave−one−out sensitivity analysis for
'exposure' on 'BDY'
